# Supplementary material for: Non-Polio Enterovirus C Replicate in Both Airway and Intestine Organotypic Cultures
Source: Viruses. 2023 Aug 27;15(9):1823. doi: 10.3390/v15091823 (PMC10537321; doi:10.3390/v15091823)
Supplement: Supplementary file 1 [file viruses-15-01823-s001.zip › viruses-2579200-supplementary.pdf]

Supplementary Figures

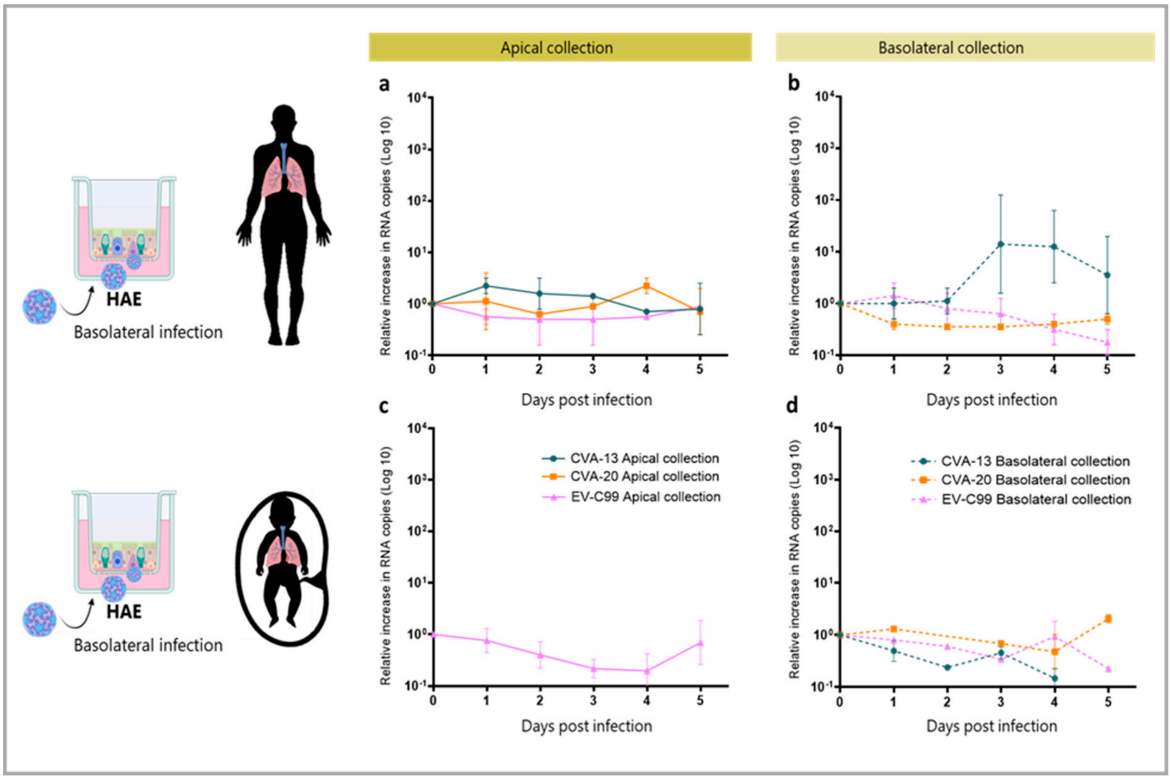

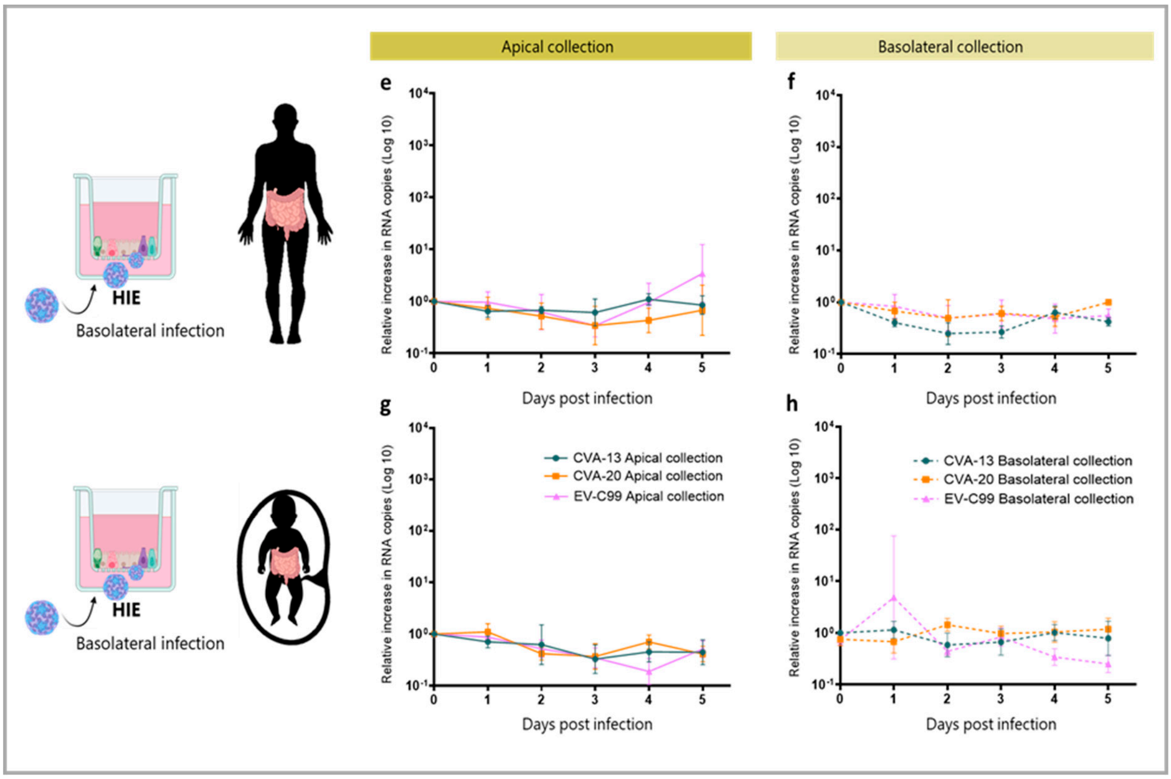

Supplementary Figure S1 | Replication of CVA-13, CVA-20, and EV-C99 on fetal and adult-derived airway and intestinal monolayers when basolaterally infected. Viral load, plotted as Log10, of CVA-13, CVA-20, and EV-C99 detected in supernatant samples by RT-qPCR and expressed as relative increase in RNA copies compared to time 0h. **(a)** Replication kinetics in the apical compartment upon basolateral infection of adult-derived airway monolayer. **(b)** Replication kinetics in the basolateral compartment upon basolateral infection of adult-derived airway monolayer. **(c)** Replication kinetics in the apical compartment upon basolateral infection of fetal-derived airway monolayer. **(d)** Replication kinetics in the basolateral compartment upon basolateral infection of fetal-derived airway monolayer. **(e)** Replication kinetics in the apical compartment upon basolateral infection of adult-derived intestinal monolayer. **(f)** Replication kinetics in the basolateral compartment upon basolateral infection of adult-derived intestinal monolayer. **(g)** Replication kinetics in the apical compartment upon basolateral infection of fetal-derived intestinal monolayer. **(h)** Replication kinetics in the basolateral compartment upon basolateral infection of fetal-derived intestinal monolayer. Data represent mean ± SEM of two technical replicates of HAE derived from a pool of 14 donors (panel a) and of three biological replicates (panel b, c, d) with three technical replicates for each. The solid line indicates replication in the apical compartment (apical collection) and dotted line indicates replication in the basolateral compartment (basolateral collection) of CVA-13 (blue), CVA-20 (orange), and EV-C99 (purple).

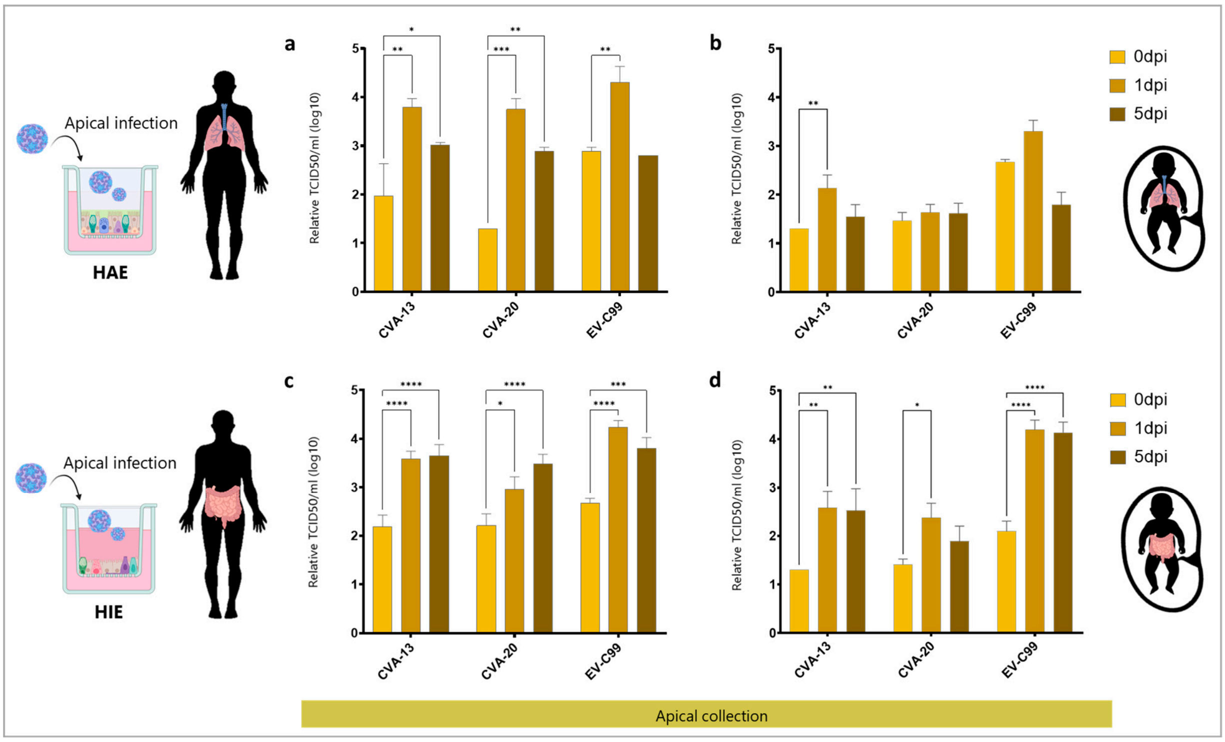

Supplementary Figure S2 | Viral titer detected in apically collected samples upon apical infection at 0h, 24h, and 120h post-infection. **(a)** Viral titer detected in infected adult-derived airway monolayer. **(b)** Viral titer detected in infected fetal-derived airway monolayer. **(c)** Viral titer detected in infected adult-derived intestinal monolayer. **(d)** Viral titer detected in infected fetal-derived intestinal monolayer. Data represent mean ± SEM of two technical replicates of HAE derived from a pool of 14 donors (panel a) and of three biological replicates (panels b, c, d) with three technical replicates each. **dpi** (days post infection), ***** p-value < 0.05, ****** p-value < 0.01, ******* p-value < 0.001, ******** p-value < 0.0001.

Supplementary Table S1 | List of antibodies used for immunostaining of HAE and HIE.

Primary Antibody (origin)	Company	cat. #	Marker	Dilution
epcam (goat)	R&D Systems	AF960	Tight junctions	1:100
zo-1 (rabbit)	Thermo Fisher Scientific	600-401-GU7	Tight junctions	1:100
**HAE**				
muc5AC - Alexa Fluor 555 conjugated (rabbit)	Abcam	ab218714	Goblet cells	1:100
β-tubulin - Alexa Fluor 647 conjugated (rabbit)	Abcam	ab204034	Ciliated cells	1:100
p63 (goat)	R&D Systems	AF1916	Basal cells	1:100
**HIE**				
villin (rabbit)	Sigma-Aldrich	346R-14	Enterocytes	1:100
chgA (goat)	Thermo Fisher Scientific	PA5-18527	Enteroendocrine cells	1:100
PCNA (rabbit)	Sigma-Aldrich	07-2162	Proliferating cells	1:100
**Secondary Antibody (origin)**	**Company**	**cat. #**	**Marker**	**Dilution**
anti-goat Alexa Fluor 488 (donkey)	Thermo Fisher Scientific	A11055	-	1:500
anti-mouse Alexa Fluor 488 (donkey)	Thermo Fisher Scientific	A21202	-	1:500
anti-goat Alexa Fluor 546 (donkey)	Thermo Fisher Scientific	A10036	-	1:500
anti-rabbit Alexa Fluor 647 (donkey)	Thermo Fisher Scientific	A31573	-	1:500
anti-goat Alexa Fluor 680 (donkey)	Thermo Fisher Scientific	A21084	-	1:500

Supplementary Table S2 | Summary of infection and shedding route for CVA-13, CVA-20, and EV-C99 replication in adult and fetal HAE and HIE.

Virus	Model	Infection route	Shedding route (medium collection)	Fold increase
**CVA-13**	HAE Adult	apical	apical	1000
	HAE Adult	apical	basolateral	100 - 1000
	HAE Fetal	apical	apical	10 - 100
	HAE Fetal	apical	basolateral	no virus detected
	HIE Adult	apical	apical	100
	HIE Adult	apical	basolateral	no virus detected
	HIE Fetal	apical	apical	10-100
	HIE Fetal	apical	basolateral	no virus detected
**CVA-20**	HAE Adult	apical	apical	1000
	HAE Adult	apical	basolateral	100 - 1000
	HAE Fetal	apical	apical	10
	HAE Fetal	apical	basolateral	no virus detected
	HIE Adult	apical	apical	100
	HIE Adult	apical	basolateral	no virus detected
	HIE Fetal	apical	apical	10 - 100
	HIE Fetal	apical	basolateral	no virus detected
**EV-C99**	HAE Adult	apical	apical	10 - 100
	HAE Adult	apical	basolateral	> 1000
	HAE Fetal	apical	apical	10
	HAE Fetal	apical	basolateral	no virus detected
	HIE Adult	apical	apical	10 - 100
	HIE Adult	apical	basolateral	no virus detected
	HIE Fetal	apical	apical	10 - 100
	HIE Fetal	apical	basolateral	10
